# School eye health services in Sri Lanka: An innovative way of approaching eye health in children

**Published:** 2017

**Authors:** Asela Abeydeera

**Affiliations:** National Coordinator, Vision 2020 Programme for Prevention of Avoidable Blindness in Sri Lanka

**Figure:**
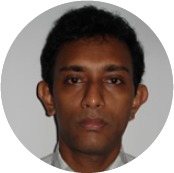
Asela Abeydeera

**The country has achieved remarkable progress in health sector domains of the millennium development goals compared to the peers in the South Asian region.**

**Figure:**
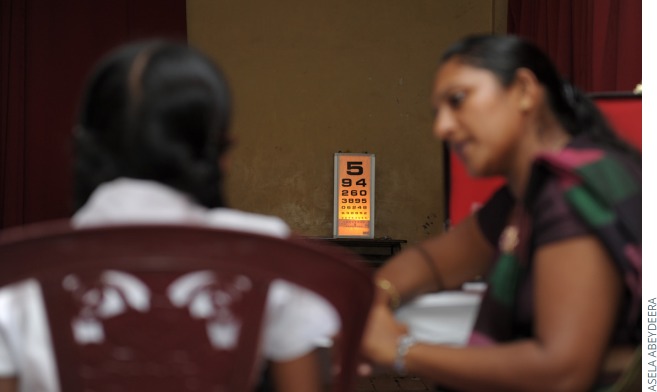
Performing refraction for a child with significant vision impairment by a volunteer optometrist. SRI LANKA

Sri Lanka, historically known as the “Pearl of the Indian Ocean,” is home to a population of 20.27 million in the year 2012 and comprised of 25 districts and nine provinces. Sri Lanka is a lower middle income country which has transitioned from an agriculture based economy towards a more urbanised economy of industry and service delivery. The country has achieved remarkable progress in health sector domains of the millennium development goals compared to its peers in the South Asian region. Further it has a unique free education and health system in the public sector, established for decades. The high literacy rate [overall 92.6% (Male 93.6% and Female 91.7%)] reflects universal access to free education system in the country. Sri Lanka provides free education, starting from primary school up to the level of post graduate higher education under public sector institutions.

## School Medical Inspection (SMI)

School children's well-being is a cornerstone of development of a country. There are 4.14 million school children in 10,162 schools in Sri Lanka. The Government of Sri Lanka spends 2.2% of GDP on education,^1^ management of human resources and development of infrastructure, centrally and, school uniforms through local governments. In addition there is a successful sponsorship programme for pupils who come from the families below poverty line. The “School Medical Inspection” (SMI) programme began in Sri Lanka in the year 1918^2^ with the objective of identifying infectious diseases among children.^3^ This has been recorded as one of the earliest such programmes, initiated globally. The SMI is a collaboration between the Ministry of Education (MOE) and Ministry of Health (MOH) through the Family Health Bureau. As part of this programme, every child entering the government school system is examined periodically for health conditions, including visual problems.

## Structure of the SMI programme

The main cadres involved in SMIs are medical officers of health (identified as MOH – a medically qualified and a registered practitioner from the public sector), public health inspectors (PHI) and public health midwives (PHM) in the local government. Based on the size of population, geographical regions have been divided among medical officers of health who work under a regional director of health services (MOH). The MOHs are responsible for conducting SMIs in their assigned areas. In the school based programme, children are examined in grade 1, 4, 7 and 10 by the SMI team. In the conventional programme, visual acuity is checked by a trained Public Health Inspector (PHI). Children who have been identified as having any visual impairment are referred to the nearest hospital with an eye clinic. At the eye clinic, children undergo refraction and prescriptions are given for spectacles. Parents have the choice of buying spectacles of their choice from private stores. It has been observed that the cost of a pair of glasses was unaffordable for most parents, especially those below the poverty line. Often at the end of the SMI process many children ended up not having appropriate vision correction with spectacles. Many limitations were observed in achieving satisfactory screening and spectacle usage under the conventional approach.

## Innovative SMI approach under the Vision 2020 programme

Having observed this scenario, the Vision 2020 Programme of the Ministry of Health – Sri Lanka initiated an action plan to to correct refractive errors in children. In this programme spectacles are issued free of charge to school children with significant refractive errors. The Vision 2020 initiative has adopted strategies locally to supply good quality spectacles, in bulk, to such children in a cost effective manner, at no cost to their parents. The results have been encouraging. The school eye health initiative is a collaboration between the Ministries of Health and Education and this programme has been successfully replicated using varied sources of non-governmental funds.

## Steps followed in the innovative approach were as follows:

1. Initial planning including identification of logistics involved, budgeting, and scheduling.
2. Signing agreements with local stakeholders, meeting regional stakeholders to schedule the project and discuss probable issues and challenges.
3. School teachers' training: Pre-screening was taught to teachers with practical illustrations in order to make them familiar with identifying children with visual impairment. A batch of approximately 50 school teachers was selected. Each teacher has the capacity to screen up to 500–1,000 children. Trained teachers were assigned to transfer skills gained to the rest of the teachers at their institution. The number of teachers to be trained was decided based on the need and if it was inadequate, higher secondary level school prefects were trained.
4. A single optotype E card was introduced to the system in order to make the pre-screening procedure easy and simple. Only a few seconds of time was spent per child for vision screening. This optotype was piloted and validated to prove its accuracy. Teachers were requested to reproduce copies of the card in similar size and quality and share with others. A Vision 2020 toll free telephone number was opened to tackle the queries they have about screening. Teachers were screening children with great success and they were listing children who had impaired vision as they were instructed. In the practical setup, it took less than half a minute to screen a child in contrast to two to three minutes with Snellen chart. Schools with lesser number of children could be screened rapidly and the schools with larger populations could be screened within two weeks.
5. Lists of children who were unable to pass through the vision test were referred to regional health authorities. Later, further examination and refraction were organised in close proximity to the MOH office or school. Mass refraction programmes were organised with participation of several optometrists and prescriptions were issued to children who had significant refractive errors (myopia or myopic astigmatism > −0.50 D sphere or cylinder or hyperopia > +0.75).
6. Children were given the choice of type, design and colour of the spectacle frame. Each child was given the best suited spectacle, which was custom made according to the prescription.
7. Eye glasses were given free of cost to those in need, with funds available from the Vision 2020 Programme of the Ministry of Health. These funds were sourced from different donor organisations. Vision 2020 programme coordinators monitored the production of good quality spectacles at a low cost. The cost of a complete pair of spectacles was around 600 LKR (4 USD), which consisted of a custom-made CR 39 lens, metal frame in the desired colour, a plastic box and a cleaning cloth.
8. Selection of suppliers for eye glasses took place annually according to tender procedures and regulations about instrument procurement under the Ministry of Health. A technical evaluation committee, comprised of eye health professionals, decided the minimum specifications of spectacles. Terms and conditions were made and an agreement was signed in order to supply spectacles as per specifications and provision of after sales services with a warranty for defects.
9. Once manufactured, spectacles were received at the head office of Vision 2020 in the capital city, a random sample was checked with an automatic lens metre prior to dispatch. The spectacles were securely sent to different regions across the country via local courier services.
10. At the district level spectacles were handed over to students through the local health and education officials with an instruction sheet to the parents on usage and care of glasses.
11. Trained teachers in each school were assigned to follow up on children who received spectacles. This included periodic checks to see whether they were using them in the correct way and to report back to the health officials with details of follow up.
12. Follow up programmes were organised in each area to replace broken and misplaced glasses and to provide new spectacles to those who needed a change in prescription.

**Figure:**
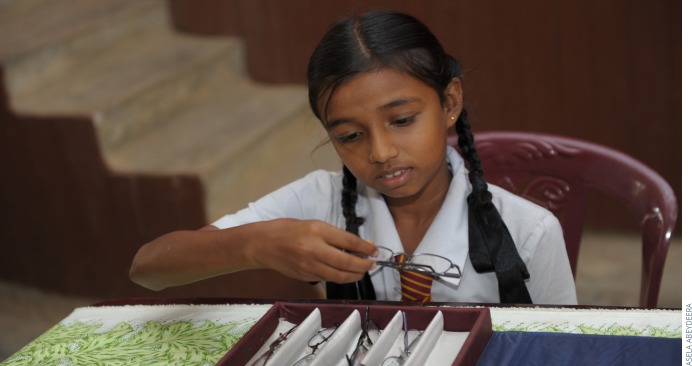
Pupils were given an opportunity to select their favourite spectacle frame. SRI LANKA

**Figure:**
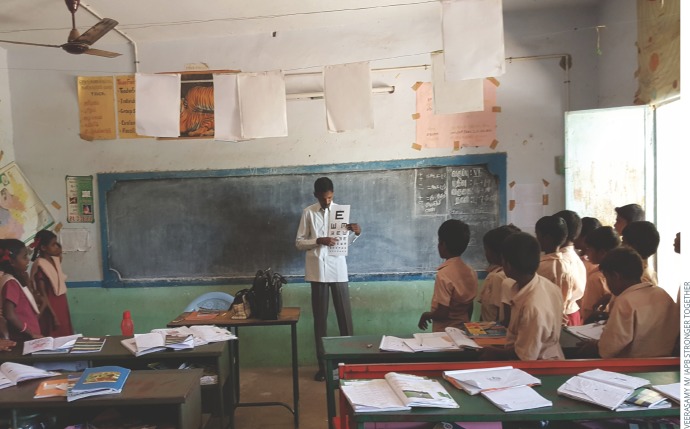
A teacher pre-screening in class. INDIA

By adapting a conventional programme in the health system and using new innovations to control avoidable blindness, the school eye health programme is a success story in Sri Lanka. This programme illustrates the possibility of achieving complete screening coverage of a targeted population using cost effective interventions. Sri Lanka could screen the entire student population and provide eye care free of charge, by integrating an eye care programme into the existing system through public-private partnerships and a multi-disciplinary approach. Several case studies from the country showed improvements in the quality of life of spectacle recipients, with many regaining better sight. Lessons learned from Sri Lanka could be useful to other neighbouring countries and the rest of the world to develop strategies to control childhood blindness.

**Highlights of Sri Lanka School Eye Screening Programme:**

- Totally free of cost for school children
- Pre-screened by trained teachers
- Funded by non-governmental organisations
- Total student population covered
- Government mediated, credibility ensured
